# Supplementary material for: Evolution of complete metamorphosis through temporal shifts in Chronologically inappropriate morphogenesis (Chinmo) and Broad
Source: Development. 2026 Jan 14;153(1):dev204998. doi: 10.1242/dev.204998 (PMC12848571; doi:10.1242/dev.204998)
Supplement: Supplementary information [file develop-153-204998-s1.pdf]

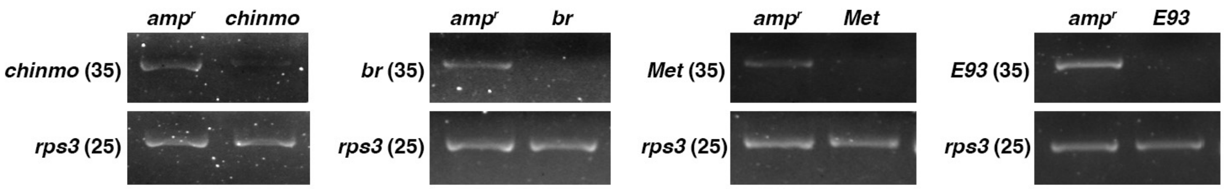

Fig. S1. Semi-quantitative RT-PCR confirmed the knockdown of *chinmo*, *br*, *Met*, and *E93*. Day 0 second instar larvae were injected with dsRNA and collected on day 3 of the third instar. *rps3* was used as a loading control. Cycle numbers for *rps3* were 22, 25, and 30. Cycle numbers for the other genes were 30, 35, and 40.

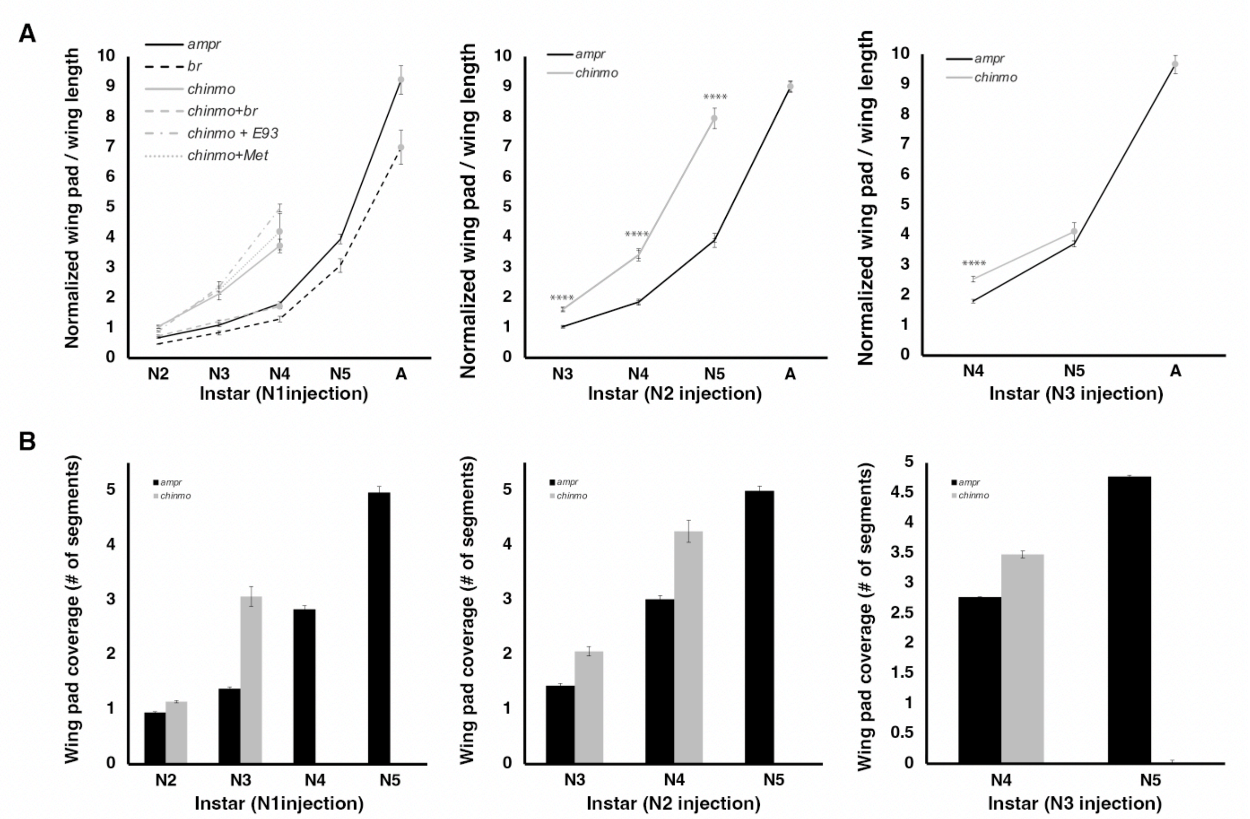

Supplemental Figure S2: Wing pad lengths increase in *chinmo* knockdown bug. (A) Effects of *br*, *chinmo*, *chinmo* + *br*, *chinmo* + *E93*, and/or *chinmo* + *Met* dsRNA injection on normalized wing pad length after dsRNA injections into first, second, and third instar nymphs. Wing lengths were normalized to the body size using the length of the fourth abdominal segment. Mean lengths with standard errors are shown. (B) Effects of *amp^r* (black bars) and *chinmo* (gray bars) dsRNA injection on wing pad coverage after dsRNA injections into first, second, and third instar nymphs. The mean number of segments that the wing pad covers and standard errors are shown.

Development • Supplementary information

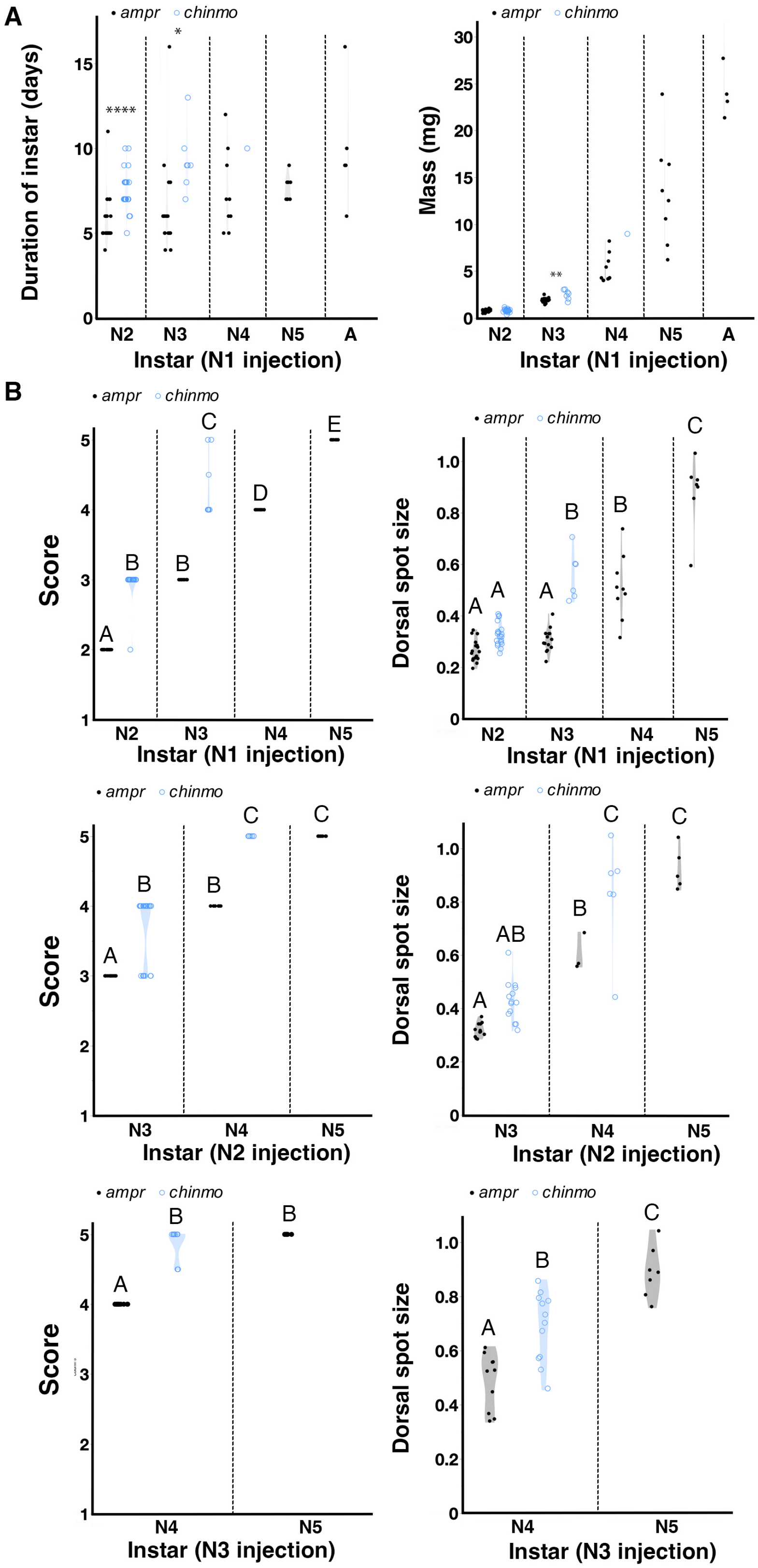

Development • Supplementary information

Fig. S3. Violin plots and individual data points for graphs shown in Fig. 3A and 3E. (A) Effect of *amp* dsRNA and *chinmo* dsRNA injection on instar duration (left) and mass (right). * represents p<0.05; ** represents p<0.01; *** represents p<0.001; **** represents p<0.0001. (B) Effect of *chinmo* dsRNA injection on the dorsal cuticular patterning. (Left) Thoracic cuticular patterns after *amp*^{r} (black dots) and *chinmo* (blue open circles) dsRNA injection in first, second and third instar nymphs. Scores based on the system shown in Fig. 3B. (Right) Normalized dorsal anterior spot size after *amp*^{r} (black dots) and *chinmo* (blue open circles) dsRNA injection in first, second and third instar nymphs. Different letters indicate statistically significant differences according to Tukey HSD.

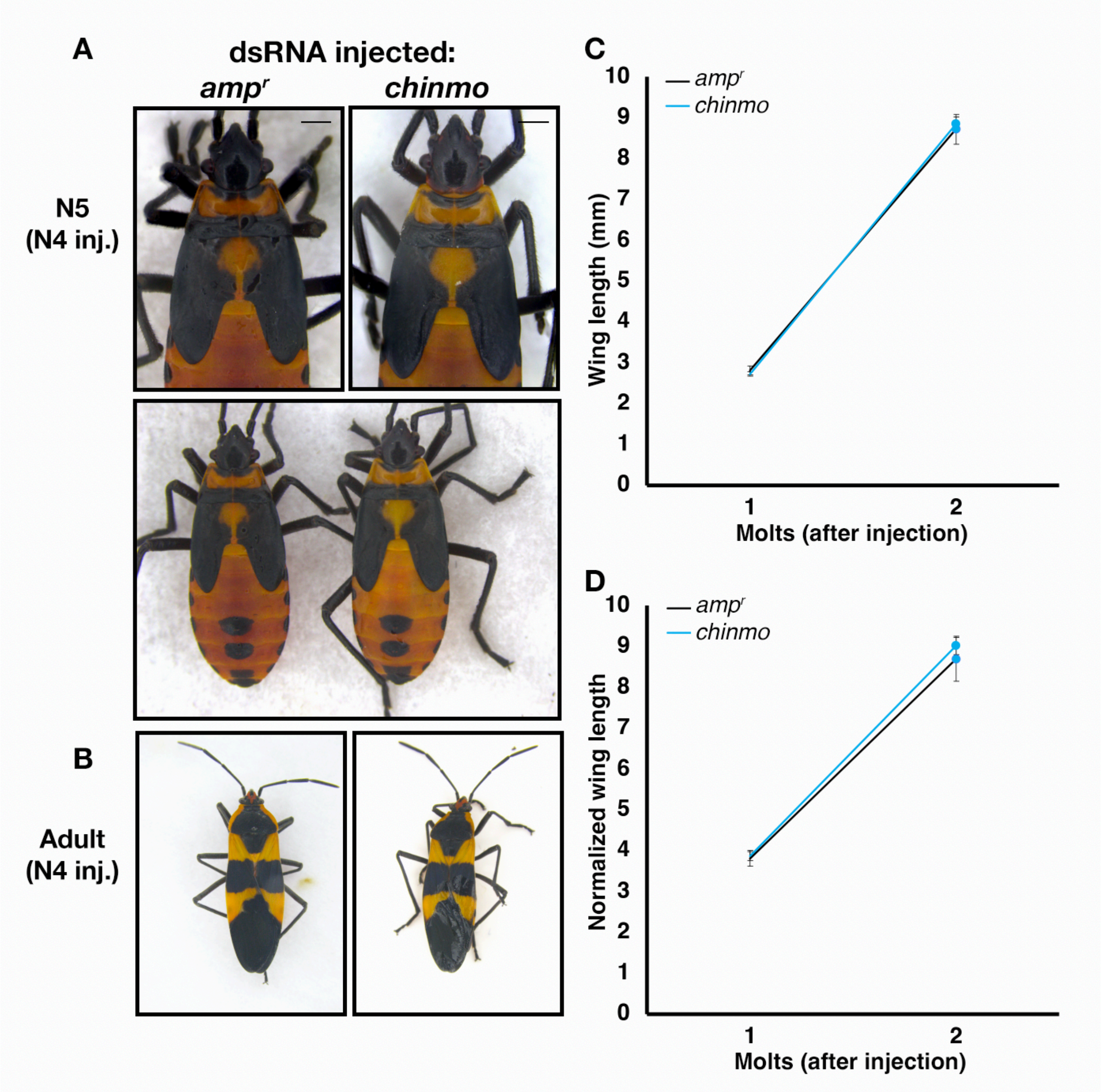

Fig. S4. *chinmo* dsRNA injection into fourth instar nymphs did not lead to more advanced patterning or precocious adulthood. (A) Both *amp*^{r} dsRNA-injected nymphs and *chinmo* dsRNA-injected nymphs molted into typical fifth instar nymphs. Side-by-side comparison shows *amp*^{r} (left) and *chinmo* (right) dsRNA-injected nymphs after one molt. Scale bars represent 0.5 mm. (B) Adult phenotypes of *amp*^{r} dsRNA-injected adult (left) and *chinmo* dsRNA-injected adult (right). (C) Wing lengths of *amp*^{r} dsRNA-injected N5 nymph and adult, and *chinmo* dsRNA-injected N5 nymph and adult. (D) Normalized wing lengths of *amp*^{r} dsRNA-injected N5 nymph and adult, and *chinmo* dsRNA-injected N5 nymph and adult. Error bars represent standard error.

Development • Supplementary information

Table S1. Primer sequences used in this study.

Available for download at
https://journals.biologists.com/dev/article-lookup/doi/10.1242/dev.204998#supplementary-data

Table S2. Phenotypes observed after dsRNA injection. First and second instars were injected until the legs stretched out.

dsRNA injected	Amount injected	Instar injected	Total # injected	Total died as nymph	Died after 1 molt	Died after 2 molts	Died after 3 molts	Died after 4 molts	Total survived to adult stage	Molted into adults after 1 molt	Molted into adults after 2 molts	Molted into adults after 3 molts	Molted into adults after 4 molts	Molted into adults after 5 molts
amp^r		**1**	**22**	17	6	6	2	3	**5**					5
amp^r		**2**	**10**	6	3		3		**4**				4	
amp^r	1 ug	**3**	**12**	5	4	1			**7**			7		
amp^r	2 ug	**4**	**6**	0	0	0			**6**		6			
chinmo		**1**	**30**	26	19	7			**4**			4		
chinmo		**2**	**22**	16	9	6	1		**6**			6		
chinmo	1 ug	**3**	**11**	7	7				**4**		4			
chinmo	2 ug	**4**	**9**	2	2				**7**		7			
br		**1**	**12**	5	1	3		1	**7**					7
E93	2 ug	**4 (day 2)**	**3**	3	1	2								
chinmo + *br*		**1**	**12**	8	6	2			**4**			4[b]		
chinmo + *E93*		**1**	**12**	11	1	7	3		**1**			1		
Met		**1**	**8**	7[c]	3	4								1
chinmo 1 + *Met* 0.5		**1**	**17**	15	8	6			**3**		1[b]	2		
chinmo 1 + *br* 1 + *Met* 0.5		**1**	**9**	1	1				**8**		8[b]			

[a] Showed adult-like pronotal patterning but could not molt completely (pharate adult).

[b] Developed into nymphal-adult intermediate.

[c] One animal molted into a normal fourth instar nymph but was not tracked beyond that stage.

Table S3. Results of Tukey HSD test following ANOVA for wing lengths and normalized wing lengths at each instar after dsRNA injection in first instar *O. fasciatus* nymphs. Different letters represent significant differences.

dsRNA injected	N2 wing length	N2 normalized wing	N3 wing length	N3 normalized wing	N4 wing length	N4 normalized wing	N5 wing length	N5 normalized wing
amp[r]	AB	A	A	A	A	A	A	A
Br	B	B	A	A	B	B	B	B
chinmo	C	C	B	B	C	C		
chinmo + br	AD	AD	A	A	AB	AB		
chinmo + e93	CD	CD	B	B	D	D		
chinmo + met	C	C	B	B	D	CD		

Table S4. Results of Tukey HSD test following ANOVA for A4 segment lengths after dsRNA injection in first instar *O. fasciatus* nymphs. Different letters represent significant differences.

dsRNA injected	N2 A4 length	N3 A4 length	N4 A4 length	N5 A4 length
amp[r]	A	A	A	A
Br	B	A	A	A
chinmo	C	A	A	
chinmo + br	A	A	A	
chinmo + e93	AC	A	A	
chinmo + met	AC	A	A	

Development • Supplementary information

Table S5. Mean weight and wing length after dsRNA treatment in fourth instar *O. fasciatus* nymphs.

dsRNA injected	Mean±SEM M1 Weight (mg)	Mean±SEM M2 Weight (mg)	Mean±SEM M1 Wing Length (mm)	Mean±SEM M2 Wing Length (mm)
*amp^r^*	17.6±0.909	41.0±3.41	2.81±0.115	8.69±0.535
chinmo	14.9±0.950	34.8±1.97	2.72±0.054	9.02±0.228
	ANOVA, F=4.7237, p=0.0488	ANOVA, F=2.66646, p=0.131	ANOVA, F=0.66146, p=0.432	ANOVA, F=0.11653, p=0.740
